# Supplementary material for: Pycea: a scverse toolkit for single-cell lineage tracing analysis
Source: bioRxiv. 2026 Nov 1:2026.10.30.763227. Preprint. [Version 1] doi: 10.64898/2026.10.30.763227 (PMC13734084; doi:10.64898/2026.10.30.763227)
Supplement: Supplementary file 1 [file NIHPP2026.10.30.763227v1-supplement-1.pdf]

bioRxiv preprint doi: https://doi.org/10.64898/2026.10.30.763227; this version posted November 1, 2026. The copyright holder for this preprint (which was not certified by peer review) is the author/funder, who has granted bioRxiv a license to display the preprint in perpetuity. It is made available under a CC-BY 4.0 International license.

Supplementary Information

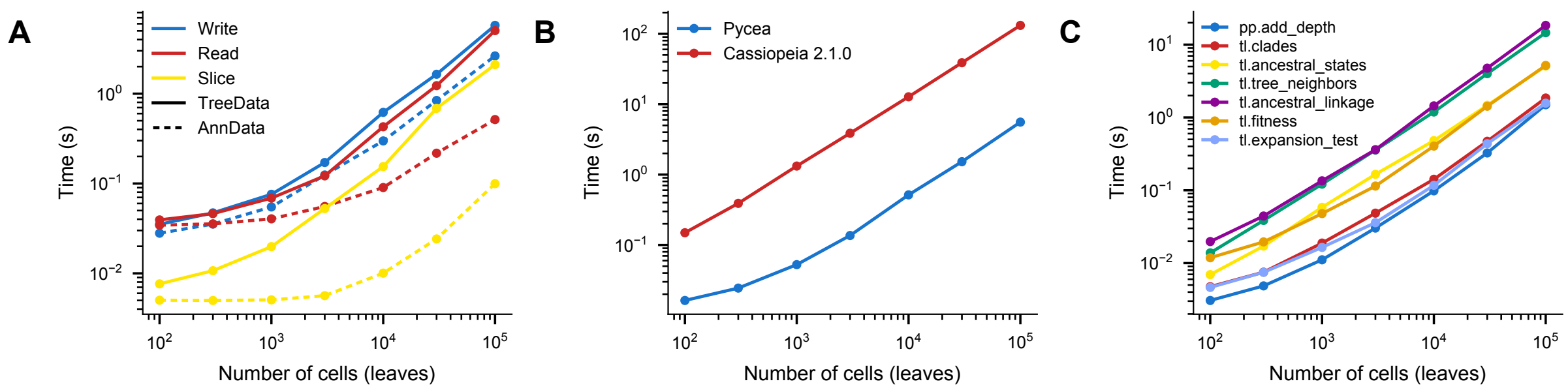

Figure S1: Benchmarks. (A) Time to write, read and slice (a random 10% of cells) TreeData and AnnData objects with a simulated lineage tree and simulated expression of 20,000 genes (sparse counts), by number of cells. **(B)** Time to plot a tree with Pycea and with Cassiopeia 2.1.0 (plotting deprecated in version 3.0.0), by number of cells. **(C)** Run time of key Pycea functions, by number of cells. Median of three runs, on a single node.

bioRxiv preprint doi: https://doi.org/10.64898/2026.10.30.763227; this version posted November 1, 2026. The copyright holder for this preprint (which was not certified by peer review) is the author/funder, who has granted bioRxiv a license to display the preprint in perpetuity. It is made available under a CC-BY 4.0 International license.

Function	Description
**pycea.pp**: Preprocessing	
add_depth	Add a depth attribute (e.g., time) to each node of the tree
**pycea.tl**: Tools	
ancestral_linkage	Measure relatedness between categories of cells
ancestral_states	Reconstruct internal node states (e.g., parsimony [39–41])
autocorr	Calculate Moran's I or Geary's C over a neighbor graph [38]
clades	Assign cells and nodes to clades
compare_distance	Compare distances between the same pairs of cells
distance	Compute distances between cells in any embedding
expansion_test	Test subclades for expansion under neutral growth [43]
fitch_count	Count transitions between states [44]
fitness	Estimate the relative fitness of each node [37]
n_extant	Count extant lineages over time
neighbor_distance	Aggregate distances from each cell to its neighbors
parsimony	Compute the small-parsimony score of a tree
partition_test	Compare the leaves below a node with its siblings or the rest
sort	Reorder branches by a node attribute
tree_distance	Compute distances between cells in the tree
tree_neighbors	Identify the closest relatives of each cell in the tree
**pycea.pl**: Plotting	
ancestral_linkage	Plot ancestral linkage as a clustered heatmap
annotation	Plot leaf annotations (e.g., metadata, expression) beside a tree
branches	Plot the branches of a tree
n_extant	Plot the number of extant lineages over time
nodes	Plot the nodes of a tree
tree	Plot a tree with branches, nodes, and annotations
**pycea.get**: Data access	
edge_df	Get tree edge attributes as a data frame
leaves	Get the leaves of a tree
node_df	Get tree node attributes as a data frame
palette	Get the color palette for a categorical variable
root	Get the root of a tree
**pycea.datasets**: Example datasets	
colgan26	Mouse embryo lineage tracing, E7.5–E10.0 [2]
koblan25	Spatial lineage tracing of 4T1 tumors [15]
packer19	*C. elegans* lineage tree and cell states [35]
yang22	KP mouse model lineage tracing [43]
yu26	DNA Typewriter mouse lineage tracing [1]

Table S1: Public functions of Pycea v0.4.0. The 33 functions in the Pycea modules. See the Pycea API documentation for details.
